# High correlation of Middle East respiratory syndrome spread with Google search and Twitter trends in Korea

**DOI:** 10.1038/srep32920

**Published:** 2016-09-06

**Authors:** Soo-Yong Shin, Dong-Woo Seo, Jisun An, Haewoon Kwak, Sung-Han Kim, Jin Gwack, Min-Woo Jo

**Affiliations:** 1 Department of Biomedical Informatics, Asan Medical Center, Seoul, Korea; 2 Department of Emergency Medicine, Asan Medical Center, University of Ulsan College of Medicine, Seoul, Korea; 3 Qatar Computing Research Institute, Hamad Bin Khalifa University, Doha, Qatar; 4 Department of Infectious Diseases, Asan Medical Center, University of Ulsan College of Medicine, Seoul, Korea; 5 Center for Disease Control and Prevention, Osong, Chungbuk, Korea; 6 Department of Preventive Medicine, University of Ulsan College of Medicine, Seoul, Korea

## Abstract

The Middle East respiratory syndrome coronavirus (MERS-CoV) was exported to Korea in 2015, resulting in a threat to neighboring nations. We evaluated the possibility of using a digital surveillance system based on web searches and social media data to monitor this MERS outbreak. We collected the number of daily laboratory-confirmed MERS cases and quarantined cases from May 11, 2015 to June 26, 2015 using the Korean government MERS portal. The daily trends observed via Google search and Twitter during the same time period were also ascertained using Google Trends and Topsy. Correlations among the data were then examined using Spearman correlation analysis. We found high correlations (>0.7) between Google search and Twitter results and the number of confirmed MERS cases for the previous three days using only four simple keywords: “MERS”, “
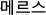
” (“MERS (in Korean)”), “
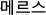
” (“MERS symptoms (in Korean)”), and “
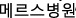
” (“MERS hospital (in Korean)”). Additionally, we found high correlations between the Google search and Twitter results and the number of quarantined cases using the above keywords. This study demonstrates the possibility of using a digital surveillance system to monitor the outbreak of MERS.

The new millennium began with the emergence of communicable diseases. In 2002, Severe Acute Respiratory Syndrome (SARS) was found in mainland China and spread throughout the world in a matter of months, with locations of incidence including Hong Kong, Taiwan, Singapore, Canada and many other countries^1^. A 2009 pandemic of H1N1spread from Mexico and was subsequently identified in the United States, Canada and globally^2^.

Middle East Respiratory Syndrome (MERS) was first reported in a patient who presented with severe respiratory illness in a hospital in Jeddah, Saudi Arabia, on June 13, 2012 and died 11 days later^3^. The virus was later isolated as a new coronavirus and named Human Coronavirus-Erasmus Medical Center (HCoV-EMC) and subsequently renamed MERS-CoV according to a global consensus^4^. Dromedaries are hosts for this virus, and there is some evidence of direct or indirect zoonotic transmission to humans. MERS is a highly fatal respiratory disease: a total of 1,782 cases and 634 deaths were reported in 27 countries as of July 2016^5^.

The outbreak in South Korea was triggered by one imported case. This outbreak caused 186 laboratory-confirmed infections, including 38 (20%) deaths as of December 22 2015, which resulted in a global threat to neighboring nations, such as China, Hong Kong, Taiwan, and Japan^6^. MERS is listed as one of the top emerging diseases likely to cause a major epidemic^7^. Importantly, MERS is considered a healthcare-associated infection; however, the exact mode of transmission remains unknown. Therefore, it is important to develop a surveillance system for detecting, tracking, reporting, and responding to MERS^8^. To enable the earlier identification of an outbreak of an emerging communicable disease such as MERS, a syndrome surveillance method that uses real-time data, including both health-related and non-health-related data, has been proposed^9^. Recently, digital surveillance approaches using non-healthcare sources, such as search engines, were developed and confirmed as a valid and useful means for identifying influenza outbreaks in real time based on several studies in the United States, European countries, Canada, New Zealand and Korea^10^,^11^,^12^,^13^,^14^,^15^,^16^.

The present study examines the correlations among social media and search engine data and the number of confirmed MERS cases and quarantined cases to evaluate the possibility of digital surveillance using a search engine and Twitter data for monitoring the outbreak of MERS.

## Results

**Figure 1:**
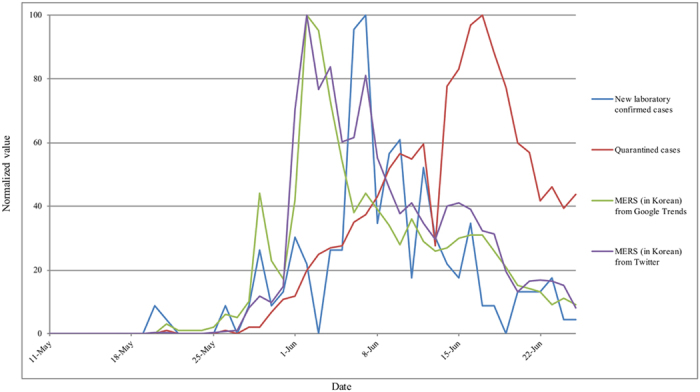
Trends of representative keywords “MERS (in Korean)” (“
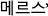
”) obtained via Google search and Twitter, the number of new laboratory-confirmed MERS cases, and the number of quarantined cases. The data are normalized to the maximum value of each dataset.

The overall trends are shown in Fig. 1, including the representative keywords “
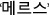
” (“MERS (in Korean)”) obtained via Google search and Twitter, the number of new laboratory-confirmed cases, and the number of quarantined cases. Peaks on Google search and Twitter with regard to use of the “MERS (in Korean)” search term are shown for June 2. New confirmed cases peaked 5 days later (i.e., June 7) and quarantined cases peaked 15 days later (i.e., June 17). In addition, overall graph patterns among them were similar. The raw data in Fig. 1 are shown in Supplementary Table 1.

**Figure 2:**
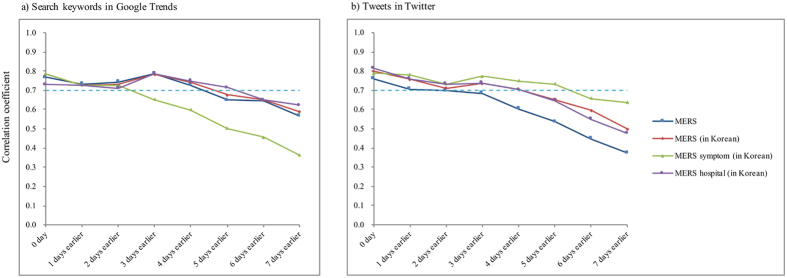
Lag correlations between new laboratory-confirmed cases of Middle East respiratory syndrome and (**a**) search keywords in Google and (**b**) tweets on Twitter.

Figure 2 and Table 1 show high lag correlations between the laboratory-confirmed cases of MERS-CoV and the Google search results (Fig. 2a) and tweets on Twitter (Fig. 2b). Three days earlier, the results obtained using the three keywords “MERS”, “MERS (in Korean)”, and “
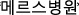
” (“MERS hospital (in Korean)”) in Google search showed high correlations (*r* > 0.7). These three keywords maintained high correlations until the four day time-lag; however, “
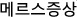
” (“MERS symptoms (in Korean)”) had the highest correlation (*r* = 0.786, *p* < 0.05) at a zero day time-lag, and this high correlation was preserved for two days. The trends for the comparisons with Twitter data were similar to those of the Google search data with high correlations and maintenance. “MERS symptoms (in Korean)” was high, but the correlation of “MERS” began decreasing from the start, similar to the results of “MERS symptoms (in Korean)” in the Google search data.

**Figure 3:**
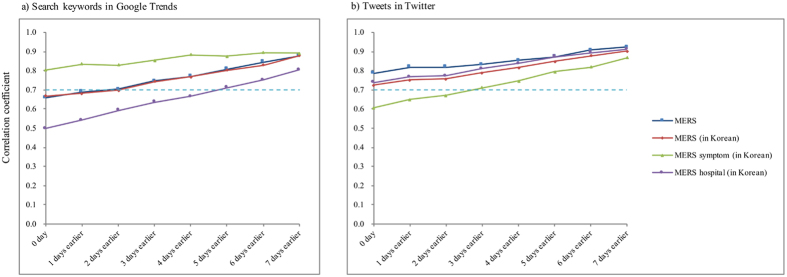
Lag correlations between the number of quarantined cases and (**a**) the search keywords in Google and (**b**) tweets on Twitter.

Figure 3 and Table 1 also show the high lag correlations between the number of quarantined cases and Google search results or Twitter tweets. However, there were some differences in the trends of the results of the quarantined cases. Contrary to the results of the new laboratory-confirmed cases, the lag correlation coefficients of all keywords continuously increased for both the Google search and Twitter results. The highest correlation coefficients of these keywords were approximately 0.9 for seven days, and they were higher than those of the new laboratory-confirmed cases. The rank of correlation for the keywords was different according to the type of cases (refer to Figs 2 and 3) and the Google/Twitter results (refer to a) and b) in Fig. 3). For example, “MERS symptoms (in Korean)” had the highest correlation coefficient with new laboratory-confirmed cases on Twitter (Fig. 2b) and with quarantined cases on Google search (Fig. 3a) but the lowest correlation coefficient for new laboratory-confirmed cases on Google (Fig. 2a) and quarantined cases on Twitter (Fig. 3b). The raw data for Figs 2 and 3 are shown in Table 1.

**Figure 4:**
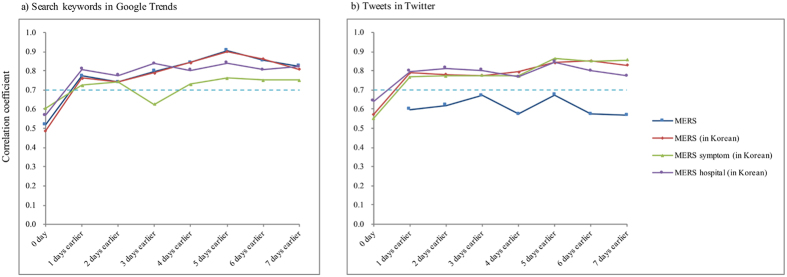
Lag correlations between new laboratory-confirmed cases of Middle East respiratory and (**a**) the search keywords in Google and (**b**) tweets on Twitter from June 3, 2015 to June 25, 2015.

The subgroup analyses focusing on the new laboratory-confirmed cases of the acceleration and deceleration period are shown in Fig. 4. Most correlation coefficients are higher than 0.7 and are maintained highly during this time lag. The ranking of correlation coefficients of the keywords during this period was similar to that of the entire study period. The results of the other subgroup analyses are provided in Supplementary Figures 1–3.

Correlation coefficients between the search keywords and tweets were high but peaks among them were somewhat different. Among the search keywords in Google, correlation coefficients were higher than 0.8, but the correlation coefficient between “MERS symptoms (in Korean)” and “MERS hospital (in Korean)” was 0.792 (Supplementary Table 2). The peak of “MERS symptoms (in Korean)” was on June 2 but the other keywords had peaks on June 2 or 3 (Supplementary Table 1). In Twitter, all correlation coefficients were higher than 0.9 except for that between “MERS” and “MERS symptoms (in Korean)” (*r* = 0.871, *p* < 0.05). The peaks of “MERS” and “MERS (in Korean)” are shown on June 2 but peaks of “MERS symptoms (in Korean)” and “MERS hospital (in Korean)” on June 7.

## Discussion

This study showed high correlations between the results obtained by searching for MERS-related keywords using Google search and Twitter and the number of confirmed MERS cases. These high correlations occurred four days before case confirmation and provide evidence that digital surveillance using a search engine and Twitter data is useful for monitoring the outbreak of an emerging infectious disease.

Because MERS is a healthcare-associated infection, it is interesting that digital surveillance using Google search and Twitter, which operate via input from the general public, may also work well for surveillance. In fact, most digital surveillance has been used to detect community-based transmitted diseases^10^,^16^,^17^,^18^,^19^,^20^. It may be related to the fact that MERS also infects the general population, such as patient family members and caregivers.

The digital surveillance methodology used in this study found increases in searches or tweets three days prior to laboratory confirmations. Generally, because a confirmatory laboratory test takes one or two days^6^,^21^,^22^, the real lag time could be one or two days. Social media and search engine data may reflect the actual disease outbreak earlier than conventional surveillance because many people use Internet searches to obtain health information before visiting a doctor^10^,^23^,^24^. The World Health Organization suggests that various factors, including lack of awareness among people and suboptimal infection and control measures, could contribute to the outbreak of MERS in Korea^25^. Given uncertain conditions associated with emerging diseases, this availability of earlier information for monitoring infectious disease will be helpful for making decisions related to disease control. Moreover, it is worth noting that important epidemiological data regarding the Korea MERS outbreak were published based on media data using digital surveillance systems by scientists in other countries. The preliminary epidemiologic assessment of the MERS outbreak in Korea was performed by Hong Kong scientists^26^, the probable transmission chains were determined by Hong Kong scientists^27^, the estimated fatality rate was determined by Japanese scientists^28^, and the risk factors for mortality were assessed by US scientists^29^. These studies emphasize the importance of rapid communication and analysis in emerging infectious diseases, and epidemiologic analyses based on media data may be a useful tool to elucidate the characteristics of ongoing outbreaks. Subgroup analyses show that a digital surveillance system could be more helpful for monitoring the spread of an emerging infectious disease than for detecting its outbreak because the correlation coefficients of the acceleration and deceleration period were higher than those of the initiation (pre-acceleration) period and all other periods. In addition, these correlations were highly maintained during the time lag. This suggests that monitoring is effective during disease spread.

Interestingly, the ranking of keywords was different according to the type of cases and Google/Twitter results. These differences may be due to various user behaviors associated with social network services versus web searches. Generally, a web search is performed to find information, whereas Twitter is used to share information with people. If people experience MERS-like symptoms, such as fever, cough, or sputum, they generally want to determine whether they have MERS; therefore, they used a web search engine, such as Google. Thus, the correlation coefficient of “MERS symptoms (in Korean)” in the quarantined cases was highest in the results from the Google search because those people may be searching “what are MERS symptoms?” However, in using Twitter, people may be attempting to deliver this information to other people. In addition, in an early phase such as the acceleration period of an unknown emerging disease like MERS, people may want to know “what is a MERS?”. Therefore, terms such as “MERS” or “MERS (in Korean)” might have early peaks. Later, people may hope to learn more specific information such as symptoms or hospitals because they might want to know whether they suffer from disease-associated morbidities or inform people what places to avoid.

Based on the present study, it may be sufficient to monitor case confirmations using simple keywords, such as the name of a disease like MERS in English or Korean; the symptoms of the disease; or a particular hospital where patients with the disease are being treated. The advantages of digital surveillance using a search engine and Twitter data are that the data can be obtained earlier, more easily and at a lower cost than via conventional surveillance techniques^10^,^11^,^12^,^13^,^14^,^15^. To improve the performance of disease surveillance, consideration of a digital surveillance system is essential.

The data used, such as Twitter tweets and Google search data, might be biased. Although all Korean public tweets were considered, Twitter is not a major social network service in Korea^30^. Additionally, Google is not one of the most used search engines in Korea^31^,^32^. However, because previous studies of influenza surveillance in Korea demonstrated the possibility of digital surveillance systems using non-dominant local search engine data or Google search^14^,^15^, the proposed digital surveillance system may be sufficient. Most digital surveillance systems using a search engine and Twitter data require the choice of specific keywords. Therefore, the keywords used by the surveillance system should be updated frequently to enable accurate monitoring of the emerging disease. For example, the term “MERS” was not generally used in Korea before the MERS outbreak (Fig. 1). However, “MERS” is one of the key terms used to detect the outbreak in this study. The promptness with which new keywords are added/searched for can significantly affect the accuracy of digital surveillance methods. Therefore, as in most studies in this field, noise, such as news reports, outbreak briefs, and health information posted on the Internet, may have affected the outcomes of this study.

## Methods

### Study period and keywords

The study period was from May 11, 2015 to Jun 26, 2015. May 11, 2015 is the symptom onset day of the first laboratory-confirmed patient^6^. Nationally acquired statistical data for MERS in Korea^6^, web-search results from Google Trends^33^, and Twitter data^34^ were compared. Based on expectations regarding public interest in this topic, the top four MERS-associated keywords used in the Google search were selected. These four keywords were “MERS”, “MERS (in Korean)”, “MERS symptoms (in Korean)”, and “MERS hospital (in Korean)”. In this study, we first included the most basic keywords, “MERS” and “MERS (in Korean)”. Next, we extended the keywords such as “MERS symptoms (in Korean)” and “MERS hospital (in Korean)”, which had a value of more than 90 among correlated queries with two basic keywords (“MERS” and “MERS (in Korean)”) in Google Trends. We also conducted a subgroup analysis focusing on the acceleration and deceleration period (June 3 - June 26, 2015) by adapting the CDC intervals^35^. CDC intervals are defined as the acceleration period indicated by a consistently increasing rate of pandemic influenza cases, indicating established transmission, and the deceleration interval indicated by a consistently decreasing rate of pandemic influenza cases.

### National MERS statistical data for Korea

Statistics data for MERS in Korea are updated daily in the MERS portal by the Korean government^6^. Daily data pertaining to new laboratory-confirmed MERS cases and quarantined cases were collected from this website. Laboratory confirmation of MERS was defined as either positive real-time reverse-transcriptase polymerase chain reaction (RT-PCR) results for at least two specific genomic targets or a single positive target with the sequencing of a second target^6^,^21^,^22^. In quarantined cases, people who were exposed to a contagious disease are separated and their movements restricted in case they become sick^36^.

### Google search and Twitter data

Daily trend data associated with the selected keywords were obtained from Google Trends by setting the location parameter to “South Korea” and the time parameter to “May to Jun, 2015.” The output was provided in a csv format. Because the data obtained using Google Trends are normalized to the total Google search volume, these data are relative^33^. The total number of searches for a given term was not provided in Google Trends. We also normalized the other data such as Twitter data and national MERS statistical data to have the same value (0 to 100) by the maximum value of each data. All data are provided in the supplementary file.

The number of tweets containing one of the predefined keywords was collected through Topsy, which is a certified partner of Twitter that offers social searching and social analytics^34^. Topsy indexes every public tweet and allows users to search these from 2013 using specific keywords. This range indicates that our analysis is based on the entire set of Korean public tweets rather than small sample sizes. Spam tweets are automatically removed by Topsy. The number of tweets, including tweets with URL links, tweets without URL links, and retweets, were collected.

### Statistical analysis

Spearman correlation analyses were used to examine the correlations among social-media, search engine data, the numbers of confirmed MERS cases, and quarantined cases using the IBM SPSS Statistics software, version 20 (IBM Corp). We used lag correlation analyses to assess the temporal relationships between these data for up to 7 days. The data on new laboratory-confirmed cases and quarantine cases were moved to the right (i.e., direction to decrease gaps of date between Google search or Twitter data and new laboratory-confirmed cases or quarantined cases). Subgroup analyses for the period were conducted along the same lines. The significance level was set at *p* < 0.05.

## Additional Information

**How to cite this article**: Shin, S.-Y. *et al*. High correlation of Middle East respiratory syndrome spread with Google search and Twitter trends in Korea. *Sci. Rep*. **6**, 32920; doi: 10.1038/srep32920 (2016).

## Supplementary Material

Supplementary Information

## Figures and Tables

**Table 1:** Lag correlations between keywords and new laboratory-confirmed and quarantined cases.

	Lag days	Google search				Twitter			
		MERS	MERS_Korean	MERS symptoms_Korean	MERS hospital_Korean	MERS	MERS_Korean	MERS symptoms_Korean	MERS hospital_Korean
New laboratory-confirmed cases	0 day	0.769	0.783	0.786	0.729	0.759	0.799	0.790	0.814
	1 day earlier	0.732	0.728	0.729	0.724	0.705	0.756	0.782	0.759
	2 days earlier	0.744	0.730	0.725	0.712	0.697	0.712	0.733	0.732
	3 days earlier	0.787	0.784	0.654	0.786	0.684	0.735	0.772	0.739
	4 days earlier	0.726	0.745	0.600	0.749	0.604	0.702	0.748	0.702
	5 days earlier	0.650	0.676	0.503	0.714	0.537	0.653	0.733	0.647
	6 days earlier	0.648	0.649	0.457	0.649	0.447	0.595	0.657	0.551
	7 days earlier	0.566	0.589	0.363	0.624	0.374	0.499	0.636	0.476
Quarantined cases	0 day	0.659	0.667	0.803	0.499	0.786	0.725	0.607	0.738
	1 day earlier	0.690	0.681	0.835	0.543	0.818	0.751	0.649	0.769
	2 days earlier	0.704	0.697	0.829	0.593	0.818	0.756	0.672	0.772
	3 days earlier	0.745	0.743	0.854	0.635	0.832	0.790	0.711	0.811
	4 days earlier	0.770	0.767	0.882	0.665	0.852	0.815	0.746	0.838
	5 days earlier	0.807	0.803	0.874	0.710	0.873	0.849	0.794	0.871
	6 days earlier	0.846	0.829	0.893	0.750	0.908	0.878	0.818	0.894
	7 days earlier	0.877	0.878	0.891	0.805	0.924	0.903	0.867	0.912

^*^N/A, Not applicable.
