# Supplementary material for: High correlation of Middle East respiratory syndrome spread with Google search and Twitter trends in Korea
Source: Sci Rep. 2016 Sep 6;6:32920. doi: 10.1038/srep32920 (PMC5011762; doi:10.1038/srep32920)
Supplement: Supplementary Information [file srep32920-s1.pdf]

High correlation of Middle East respiratory syndrome spread with Google search and Twitter trends in Korea

Soo-Yong Shin, PhD[1,*], Dong-Woo Seo, MD[2,*], Jisun An, PhD[3], Haewoon Kwak, PhD[3], Sung-Han Kim, MD[4], Jin Gwack, MD[5], Min-Woo Jo, MD[6]

[1]Department of Biomedical Informatics, Asan Medical Center, Seoul, Korea

[2]Department of Emergency Medicine, Asan Medical Center, University of Ulsan College of Medicine, Seoul, Korea

[3]Qatar Computing Research Institute, Hamad Bin Khalifa University, Doha, Qatar

[4]Department of Infectious Diseases, Asan Medical Center, University of Ulsan College of Medicine, Seoul, Korea

[5]Center for Disease Control and Prevention, Osong, Chungbuk, Korea

[6]Department of Preventive Medicine, University of Ulsan College of Medicine, Seoul, Korea

* These authors contributed equally to this work.

Corresponding author: Min-Woo Jo, MD, PhD

Department of Preventive Medicine, University of Ulsan College of Medicine, 88 Olympic-ro 43 gil, Songpa-gu, Seoul 05505, Korea

Tel: +82-2-3010-4264

Fax: +82-2-477-2898

E-mail:mdjominwoo@gmail.com

Supplementary Table 1. Raw data of queries, new laboratory confirmed cases and quarantined cases.

Date	New_ confirmed	Quaran- tined	Google search				Twitter			
			G_MERS	G_MERS _Korean	G_MERS symptoms_ Korean	G_MERS hospital_ Korean	T_MERS	T_MERS _Korean	T_MERS symptoms_ Korean	T_MERS hospital_ Korean
11-May	0	0	0	0	0	0	736	1	0	0
12-May	0	0	0	0	0	0	738	5	0	0
13-May	0	0	0	0	0	0	418	1	0	0
14-May	0	0	0	0	0	0	603	3	0	0
15-May	0	0	0	0	0	0	373	4	0	0
16-May	0	0	0	0	0	0	374	2	0	0
17-May	0	0	0	0	0	0	600	9	0	0
18-May	0	0	0	0	0	0	544	1	0	0
19-May	0	0	0	0	0	0	1087	84	1	0
20-May	2	3	0	0	0	0	1711	631	19	3
21-May	1	64	0	3	0	0	1425	619	50	9
22-May	0	0	2	1	0	0	1007	119	7	5
23-May	0	0	2	1	0	0	481	112	30	1
24-May	0	0	2	1	0	0	552	86	34	4
25-May	0	0	2	2	0	0	999	984	96	83
26-May	2	61	2	6	0	0	2821	1519	81	56
27-May	0	0	3	5	0	0	3652	2307	116	74
28-May	2	127	8	10	0	15	5876	17156	1112	1618
29-May	6	129	69	44	14	21	15615	25429	1083	1542
30-May	2	462	30	23	9	25	11650	21000	960	1155
31-May	3	715	15	17	12	17	11940	31608	753	2485
01-Jun	7	789	41	42	14	55	68422	151491	4629	12847
02-Jun	5	1346	91	100	34	100	99439	215940	3543	23063
03-Jun	0	1667	100	95	34	92	92846	165605	3815	14391
04-Jun	6	1820	98	73	32	75	97356	180961	3213	24275

05-Jun	6	1866	82	54	100	52	64182	130330	3121	20499
06-Jun	22	2361	51	38	56	42	55031	132809	2629	28492
07-Jun	23	2508	52	44	54	93	73464	174900	6050	35538
08-Jun	8	2892	57	39	65	48	80942	118742	1994	18080
09-Jun	13	3493	48	34	40	46	80686	98992	1689	14448
10-Jun	14	3805	35	28	45	31	76577	81095	1424	13604
11-Jun	4	3680	42	36	49	35	81867	88904	1730	14500
12-Jun	12	4014	33	29	27	40	76244	74955	1180	8224
13-Jun	7	1856	27	26	31	36	81169	64119	1075	8450
14-Jun	5	5216	26	27	44	33	75017	86297	1415	12376
15-Jun	4	5586	36	30	32	36	86543	88754	1472	10193
16-Jun	8	6508	36	31	36	21	72566	83927	1099	7962
17-Jun	2	6729	32	31	44	28	57311	70048	956	9002
18-Jun	2	5930	28	26	29	20	74650	67781	499	9809
19-Jun	0	5197	21	21	28	17	61836	41851	238	4304
20-Jun	3	4035	16	15	15	0	37784	28378	78	3141
21-Jun	3	3833	14	14	20	0	33054	35301	92	4114
22-Jun	3	2805	16	13	14	0	30099	36025	143	6280
23-Jun	4	3103	15	9	12	0	26619	35891	189	6079
24-Jun	1	2642	15	11	15	0	23934	32517	201	3142
25-Jun	1	2931	14	9	9	0	18209	17715	44	1334
26-Jun	1	2467	9	7	12	0	12869	7059	242	1721

Supplementary Table 2. Correlations between the search keywords in Google (A) and tweets on Twitter (B)

(A) Correlations between the search keywords in Google

	G_MERS	G_MERS_Korean	G_MERS symptoms_Korean	G_MERS hospital_Korean
G_MERS	1.000	0.987	0.857	0.880
G_MERS_Korean		1.000	0.861	0.890
G_MERS symptoms_Korean			1.000	0.792
G_MERS hospital_Korean				1.000

(B) Correlations between the tweets on Twitter

	T_MERS	T_MERS_ Korean	T_MERS symptoms_Korean	T_MERS hospital_Korean
T_MERS	1.000	0.941	0.871	0.924
T_MERS_ Korean		1.000	0.946	0.979
T_MERS symptoms_Korean			1.000	0.933
T_MERS hospital_Korean				1.000
